# Pilot study on the influence of acute alcohol exposure on biophysical parameters of leukocytes

**DOI:** 10.3389/fmolb.2023.1243155

**Published:** 2023-08-08

**Authors:** Puya Shalchi-Amirkhiz, Tristan Bensch, Undine Proschmann, Ann-Kathrin Stock, Tjalf Ziemssen, Katja Akgün

**Affiliations:** ^1^ Multiple Sclerosis Center, Center of Clinical Neuroscience, Department of Neurology, University Hospital Carl Gustav Carus, Dresden University of Technology, Dresden, Germany; ^2^ Cognitive Neurophysiology, Department of Child and Adolescent Psychiatry, Faculty of Medicine of the TU Dresden, Dresden, Germany; ^3^ Biopsychology, Department of Psychology, School of Science, TU Dresden, Dresden, Germany

**Keywords:** binge drinking, alcohol, ethanol, leukocytes, immune system, cell deformability, real-time deformability cytometry, cell mechanics

## Abstract

**Objective:** This pilot study explores the influence of acute alcohol exposure on cell mechanical properties of steady-state and activated leukocytes conducted with real-time deformability cytometry.

**Methods:** Nineteen healthy male volunteers were enrolled to investigate the effect of binge drinking on biophysical properties and cell counts of peripheral blood leukocytes. Each participant consumed an individualized amount of alcohol to achieve a blood alcohol concentration of 1.2 ‰ as a mean peak. In addition, we also incubated whole blood samples from healthy donors with various ethanol concentrations and performed stimulation experiments using lipopolysaccharide and CytoStim™ in the presence of ethanol.

**Results:** Our findings indicate that the biophysical properties of steady-state leukocytes are not significantly affected by a single episode of binge drinking within the first two hours. However, we observed significant alterations in relative cell counts and a shift toward a memory T cell phenotype. Moreover, exposure to ethanol during stimulation appears to inhibit the cytoskeleton reorganization of monocytes, as evidenced by a hindered increase in cell deformability.

**Conclusion:** Our observations indicate the promising potential of cell mechanical analysis in understanding the influence of ethanol on immune cell functions. Nevertheless, additional investigations in this field are warranted to validate biophysical properties as biomarkers or prognostic indicators for alcohol-related changes in the immune system.

## 1 Introduction

Numerous studies have demonstrated the broad effects of alcohol on virtually all organ systems in the human body, including the cardiovascular system, gastrointestinal system, nervous system, and immune system (Albert et al., 1999; Belleville, 2002; Ronksley et al., 2011; de la Monte and Kril, 2014; Engen et al., 2015; Louvet and Mathurin, 2015; Pasala et al., 2015; Barr et al., 2016; Bishehsari et al., 2017; Obad et al., 2018; Le Dare et al., 2019). Interestingly, the impact of alcohol on human health and mortality appears to be dose-dependent (Barr et al., 2016). Light to moderate alcohol consumption is associated with beneficial effects on cardiovascular and autoimmune diseases (e.g., rheumatoid arthritis or hyperthyroidism) (Maxwell et al., 2010; Gil-Bernabe et al., 2011; Carlé et al., 2012; Carlé et al., 2013; Nurmi et al., 2013). On the other hand, high-dosed and chronic consumption has been linked to cardiomyopathies, increased risk of viral or bacterial infections, and a variety of cancers (e.g., pharynx, larynx, esophagus, liver) (Happel and Nelson, 2005; Baliunas et al., 2010; Li et al., 2014; Turati et al., 2014; Le Dare et al., 2019; Fernández-Solà, 2020). However, the observed effects of alcohol on the human body are complex and a topic of controversial discussion in the scientific community. Several recent studies have found growing evidence that even moderate alcohol consumption increases health risks, leading to the suggestion that the beneficial effects of alcohol depend on sex, age, background diseases, and region (Topiwala et al., 2017; Bryazka et al., 2022; Topiwala et al., 2022).

Binge drinking (i.e., high-dosage alcohol intoxication) is a harmful drinking pattern defined as alcohol consumption that elevates the blood alcohol concentration (BAC) to 0.8‰, which typically occurs after five or more drinks by men and four or more drinks by women within 2 h (National Institute on Alcohol Abuse and Alcoholism, 2004; Molina and Nelson, 2018). It leads to increased mortality and is mainly popular among young adults but also occurs in high frequency among elderly adults (Courtney and Polich, 2009; Han et al., 2019; Surial et al., 2021). Consuming large amounts of alcohol in a short period of time results in high BAC which is concerning due to several detrimental pathophysiological mechanisms that have been identified. Alcohol can permeate all organ tissues causing inflammation, oxidative stress, mitochondrial injury, and cell membrane perturbation (Molina and Nelson, 2018). Furthermore, several studies have underscored the profound impact of ethanol on the gene expression of pattern recognition receptors (PRR), activation states, cytokine expression, phagocytic activity, reactive oxygen species (ROS) release, cell counts, and migration in both innate and adaptive immune cells (Schmidt and Lint, 1972; Saitz et al., 1997; Molina et al., 2003; Molina et al., 2014; Molina and Nelson, 2018; Stadlbauer et al., 2019).

The examination of cells’ biophysical properties has attracted growing attention in recent research, as new and more feasible methods were established for cell analyzing (Gossett et al., 2012; Byun et al., 2013; Otto et al., 2015; Guillou et al., 2016; Nyberg et al., 2017). The substantial role of the cytoskeleton in essential cell functions like activation, mitosis, migration, and phagocytosis provides the opportunity to use cell mechanical characteristics as promising biomarkers for biological processes in health and disease (Di Carlo, 2012). Until recently, it was challenging to measure more than a few or several hundred cells per hour with methods like atomic force microscopy (AFM), micropipette aspiration, or optical stretcher (Binnig et al., 1986; Hochmuth, 2000; Guck et al., 2001). These techniques did not provide the means for analyzing large cohorts of cell populations in screening procedures or routine clinical usage. For this reason, among others, cell mechanical measurements took only place in basic biophysics research (Di Carlo, 2012).

While numerous studies have investigated the effect of alcohol on the cellular mechanisms of leukocytes, the impact on their mechanical properties remains largely unexplored. Recognizing ethanol-specific changes in cell mechanics could illuminate alterations in the immune constitution of individuals with acute alcohol intoxication or those diagnosed with alcohol use disorder (AUD), potentially marking these alterations as biomarkers. In response to this knowledge gap, our pilot study was designed to employ real-time deformability cytometry (RT-DC)—a novel tool for high-throughput cell mechanical analysis (Otto et al., 2015). Specifically, we examined the potential effects of acute alcohol exposure on the cell mechanics of both steady-state leukocytes *ex vivo* and activated leukocytes *in vitro*.

## 2 Materials and methods

### 2.1 Participants

Nineteen healthy male participants (mean age 26.3 ± 2.66 years) were enrolled in this study. To avoid confounding effects caused by hormonal fluctuations or the menstrual cycle, only male subjects were included in this study. Inclusion criteria for participants were the absence of neurological, psychiatric, or somatic diseases as well as any kind of medication. To exclude participants with a high risk of alcohol addiction/abuse or pronounced homeostatic alcohol tolerance, each subject had to complete the AUDIT (alcohol use disorders identification test) with a score between 1 and 15 (Babor et al., 2001). Baseline characteristics are given in Table 1. The experiments complied with the declaration of Helsinki and were done with the approval of the Ethics Board of the Medical Faculty of TU Dresden (ID: EK-348092014). All patients gave their informed consent.

**TABLE 1:** Baseline characteristics.

Parameter	Mean ± SD (n = 19)
Age (in years)	26.3 ± 2.66
Height (in cm)	180.2 ± 7.17
Weight (in kg)	76.53 ± 7.15
Sport (h per week)	4.97 ± 2.64
BAC* level in ‰	
• T0	0 ± 0
• T1	1.26 ± 0.24
• T2	1.14 ± 0.19
AUDIT*	6.6 ± 1.98

*BAC, blood alcohol concentration; AUDIT, alcohol use disorders identification test.

### 2.2 Alcohol administration, alcohol breath-testing, sample collection

The participants were asked to fast at least 3 h prior to testing and to refrain from consuming any type of stimulants (e.g., caffeine, guanine, nicotine) or sedative substances 4 h prior to alcohol administration. A version of the Widmark and Watson, Watson & Batt equation was used to calculate individualized amounts of vodka (40% alcohol by volume) for each subject (Widmark, 1932; Watson et al., 1980). The equation was incorporated into a tool, which is available in the supplementary data section of this paper and was also used by Stock et al. (2017). Considering an absorption deficit of 20%, our goal was to achieve a maximal possible BAC of 1.5‰, with a mean peak at 1.2‰ (26.04 mmol/l). Orange juice was used to dilute the vodka to a 50:50 blend and participants were asked to drink their beverage within 30 min. Alcohol breath tests were performed with the Alcotest^®^ 3000 (Drägerwerk, Lübeck, Germany) before and after (1, 2 h) alcohol administration according to the manufacturer’s instructions. In previous studies, Stock et al. (2014), Stock et al. (2016) showed that alcohol breath-testing yields the same results as the analysis of venous blood using a capillary gas chromatography-headspace technique. The breath-testing and whole blood collection was performed at the same time points (Supplementary Figure S1A).

### 2.3 Immunophenotyping by fluorescence-activated cell sorting (FACS)

Venous blood was drawn into an ethylenediaminetetraacetic acid (EDTA) S-monovette (Sarstedt, Nümbrecht, Germany) for immunophenotyping. Whole blood (100 µl per flow cytometry panel) was directly stained in FACS tubes with fluorescence-labeled antibodies (BD-Biosciences, Franklin Lakes, United States: CD8-PerCP-Cy5.5, CD5-PE, CD45RA-PE-CF594, CD19-PE-Cy5, CD4-PE-Cy7, CD45RO-APC, CD38-A700, CD3-APC-H7, CD138-BV421, CD10-BV510, CD27-BV605, CD14-PE-CF594, CD19-PE-Cy5, CD25-PE-Cy7, CD16-A700, CD3-APC-H7, CD11b-BV421, HLADR-BV510; Biolegend, San Diego, United States: CD11c-BV605; Miltenyi Biotec, Bergisch-Gladbach, Germany: BDCA1-APC, BDCA2-PE) for 15 min in the dark at room temperature (RT). After washing, red blood cell (RBC) lysis was performed with 2 ml ACK lysing buffer (Thermo Scientific, Waltham, United States) for 7 min in the dark at RT. 2 ml of FACS buffer (phosphate buffered saline, 3% fetal calf serum, 1% sodium azide) was added to wash the samples twice. Subsequently, cells were resuspended in 400 µl FACS-buffer. After the staining procedure, cells were measured by flow cytometry (LSR Fortessa cytometer, BD Biosciences, Franklin Lakes, United States) and analyzed with FACS-Diva Software (BD Biosciences, Franklin Lakes, United States). The gating strategy is available in the supplementary data section of this paper (Supplementary Figure S7).

### 2.4 Sample preparation and *ex vivo* RT-DC measurements

For *ex vivo* RT-DC measurements, venous blood was drawn from the binge-drinking participants into a sodium citrate S-monovette (Sarstedt, Nümbrecht, Germany) before and after alcohol intoxication. The measurements were performed as previously described with an AcCellerator (Zellmechanik Dresden GmbH, Dresden, Germany) (Otto et al., 2015). 50 μl of the blood sample were suspended in 950 µl (1:20 dilution) of the viscosity-adjusted CellCarrier B measurement buffer (Zellmechanik Dresden GmbH, Dresden, Germany) based on 1X PBS and methylcellulose. Two 1 ml syringes, filled with CellCarrier B, were connected to PEEK tubes (IDEX Health & Science LLC, Oak Harbor, United States) and placed on a syringe pump (neMESYS, Cetoni GmbH, Korbussen, Germany). The sample was aspirated into the sample tube, which was then connected to the sample inlet of the microfluidic chip with a square cross-section of 20 μm × 20 µm. The second tube contained a measurement buffer and was connected to the second inlet for the sheath flow. The total flow rate during the measurements was 0.08 μl/s (sheath flow rate: 0.06 μl/s; sample flow rate: 0.02 μl/s). An inverted high-speed microscope equipped with a CMOS camera was used to acquire images of the cells in the region of interest (ROI). Cells were measured in the constricted channel area where shear stress and pressure gradients lead to deformation. The biophysical parameters were displayed in ShapeIn software (Zellmechanik GmbH, Dresden, Germany) in real-time and were analyzed after the measurement using ShapeOut software (Zellmechanik GmbH, Dresden, Germany). Lymphocytes, monocytes, and neutrophils were identified separately in the cell brightness-area parameter plot as described in Toepfner et al. (2018) (Supplementary Figure S1). In brief, during the measurement, an image of each analyzed cell is saved, and the average pixel brightness is determined. The cell size, presented as the cell area (in µm^2^), and the brightness differences are enough to distinguish between these subpopulations (Toepfner et al., 2018). Importantly, the lymphocyte population encompasses natural killer (NK) cells (Otto et al., 2015). Unfortunately, at present, there is no method to distinguish between these cell populations using RT-DC. Deformation values represent a calculated, unitless metric that quantifies the deviation of a cell´s shape from circularity, while Young’s modulus is a parameter that quantifies the overall stiffness of a cell (Kubánková et al., 2021). Moreover, RT-DC measurements provide data regarding the cell area and volume, along with standard deviations (SD) for all parameters. These are denoted as SD deformation, SD cell area, SD Young’s modulus, and SD volume.

### 2.5 *In vitro* RT-DC measurements of whole blood samples

For *in vitro* measurements, blood was drawn from ten healthy donors (mean age 26.9 ± 4 years) into a sodium citrate S-monovette (Sarstedt, Nümbrecht, Germany). These samples were incubated with 26.04 mmol/l (≙ 1.2%) ethanol for 2 hours at RT. This concentration was chosen to mirror the BAC in our binge-drinking participants. Subsequently, we repeated the experiments with another set of ten healthy donors (mean age 28.1 ± 8 years) using 86.8 mmol/l (≙ 4.0%) ethanol. The higher ethanol concentration was employed in our *in vitro* studies as such levels occur in patients with chronic alcohol abuse following acute high-dosage drinking but may result in coma or death in healthy individuals (Szabo et al., 1995). Each donor provided a sample for both control and treatment measurements. In both experiments, the control group consisted of whole blood samples with phosphate-buffered saline (PBS) added. To prevent any potential dilution effects, the control and treatment groups received the same volume of either PBS or ethanol at the respective final concentrations (26.06 mmol/l or 86.6 mmol/l). The *in vitro* experiments were conducted separately for each ethanol concentration using two different sets of ten healthy donors. Consequently, the data was analyzed using individual plots for each concentration, which included both the control group and the respective ethanol-treated group.

Further, we utilized two distinct stimuli to investigate the biophysical properties of both innate and adaptive immune cells in the presence of ethanol during stimulation. Again, all measurements were conducted with whole blood samples. Our *in vitro* stimulation studies were performed with blood from healthy donors in two separate experiments with either LPS (Sigma Aldrich, St. Louis, United States) or CytoStim™ (Miltenyi Biotec, Bergisch Gladbach, Germany) for 4 h at 37°C. Monocytes and neutrophils, key components of the innate immune system, exhibit strong responsiveness to LPS via their toll-like receptor (TLR) 4 complex, leading to various changes including activation and migration, among others (Williams and Ridley, 2000; Ali et al., 2007; Kleveta et al., 2012; Jakubzick et al., 2017; Orekhov et al., 2019; Chaiwut and Kasinrerk, 2022). Capitalizing on the ability of RT-DC to analyze these LPS-responsive cells in a label-free manner, our focus in this experiment was primarily on these two subsets. However, considering the potential effects of an inflammatory milieu on the biophysical properties of lymphocytes, these cells have been analyzed, too. The experimental design (LPS: mean age 26.7 ± 7.8 years) incorporated four groups: a control group with PBS (steady-state), a stimulated group (activated), and two stimulated groups that were exposed to either 26.04 mmol/l or 86.8 mmol/l ethanol. This setup enabled us to assess, whether the cellular response to stimulation differed when cells were exposed to ethanol, compared to the control group.

T cells play a central role in the adaptive immune response and can be effectively stimulated by CytoStim™, a superantigen-like reagent, primarily composed of polyclonal antibody-based constituents (Chaplin, 2010; Campbell et al., 2011)*.* As a member of the lymphocyte subset, these cells can be analyzed with RT-DC (Otto et al., 2015). To include these crucial cells in our study, we utilized CytoStim™ in our stimulation experiments (CytoStim™: mean age 27.3 ± 7.9 years) and mirrored the experimental design of the LPS stimulation experiments. It is important to note that the presence of antigen-presenting cells (APCs), such as monocytes, is essential for the success of stimulation experiments utilizing CytoStim™ (Campbell et al., 2011). Therefore, we also evaluated monocytes. Given the potential influence of an inflammatory environment on the mechanical properties of neutrophils, these cells were also included in our analysis.

### 2.6 Statistical analysis

As demonstrated in previous RT-DC studies, the Shape Out software (Zellmechanik Dresden GmbH) yields the median values and standard deviations for deformation, cell area, Young’s modulus and volume which can be used for further statistical analysis (Toepfner et al., 2018; Kubánková et al., 2021). For immunophenotyping, mean values were used for statistical analysis as is customary. Of the nineteen participants initially recruited for this study, all were immunophenotyped. However, only thirteen could be included in the final RT-DC analysis due to technical difficulties encountered during the measurements with the applied methodology. Statistical analysis was done in GraphPad Prism version 9.4.1 for Mac (GraphPad Software, San Diego, CA, United States) using repeated measures one-way ANOVA with Dunnett *post hoc* testing or paired t-tests as indicated in the figure legends.

## 3 Results

### 3.1 Effect of acute alcohol intoxication on immune cell deformability and cell size in healthy male participants

**FIGURE 1:**
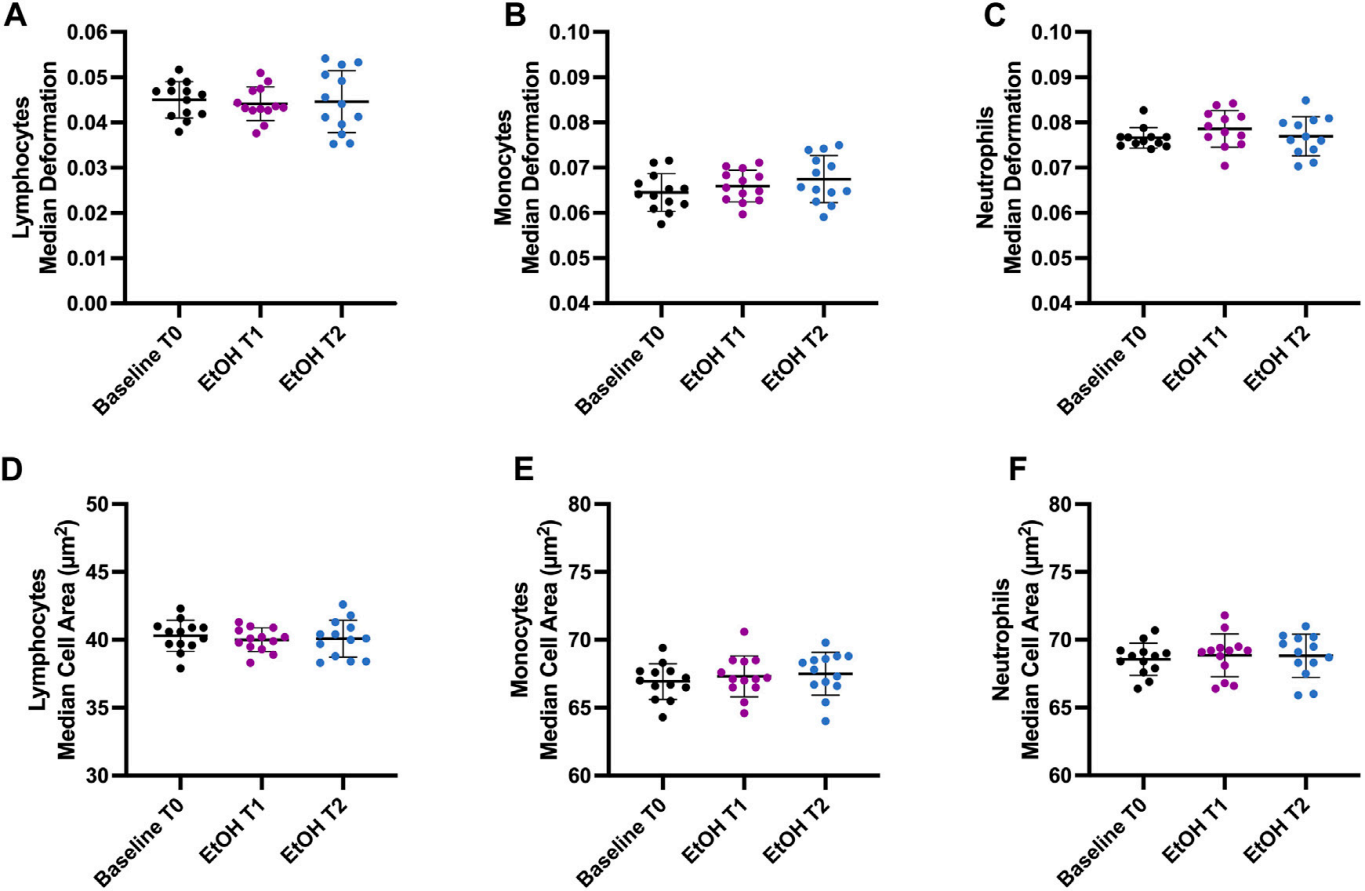
Effect of binge drinking on cell deformability and cell size. **(A–C)** Median deformation of lymphocytes **(A)**, monocytes **(B)**, and neutrophils **(C)** before (T0), 1 h (T1), and 2 h (T2) after intoxication with an alcoholic beverage (vodka with orange juice). **(D–F)** Median cell area (µm^2^) of lymphocytes **(D)**, monocytes **(E)**, and neutrophils **(F)**. T0 represents the baseline when the participants were sober. Our objective was to identify within-group differences following binge drinking. Statistical comparisons were done using repeated measures one-way ANOVA with Dunnett *post hoc* testing. One, two, three, or four asterisks indicate the significance levels *p* < 0.05, *p* < 0.01, *p* < 0.001, and *p* < 0.0001. (n = 13).

Healthy male participants were enrolled in this study to investigate the effect of binge drinking on the mechanical and morphological properties of immune cells. To identify within-group differences, we conducted measurements before binge drinking to establish baseline values (T0). We then compared these baseline measurements with those taken at the peak BAC and post-peak BAC (Supplementary Figure S1A). Lymphocytes, monocytes, and neutrophils were analyzed after RT-DC measurements. Compared to the baseline values, there were no significant changes in cell deformability, size, Young’s modulus, and volume of lymphocytes, monocytes, or neutrophils after acute alcohol intoxication (Figure 1).

### 3.2 Effect of acute alcohol intoxication on immune cell counts in healthy male participants

**FIGURE 2:**
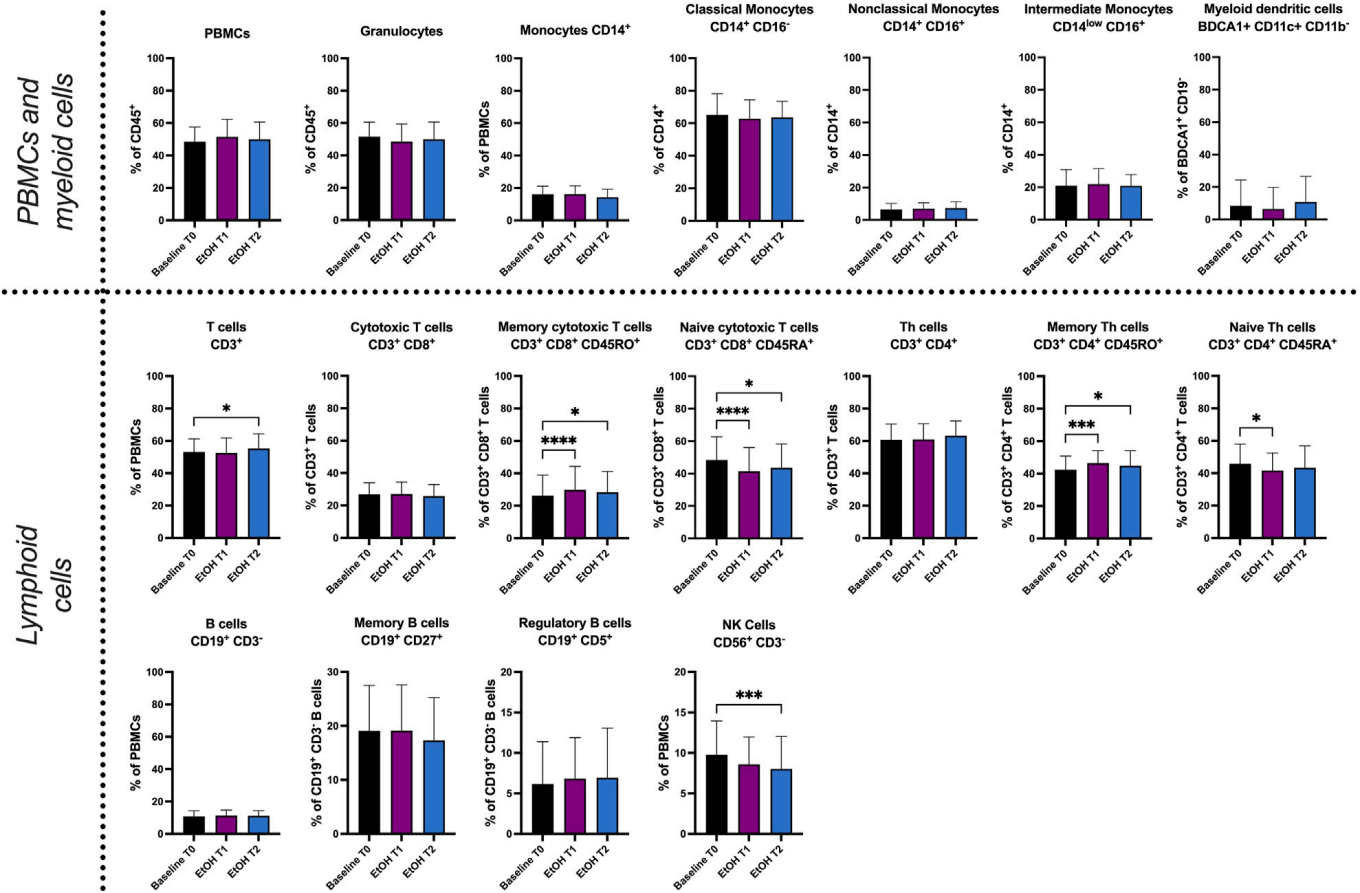
Immunophenotyping of whole blood by fluorescence activated cell sorting (FACS). Relative cell count (in %) of PBMCs, granulocytes, monocytes (CD14^+^), classical monocytes (CD14^+^ CD16^−^), nonclassical monocytes (CD14^+^ CD16^+^), intermediate monocytes (CD14^low^ CD16^+^), myeloid dendritic cells (BDCA1^+^ CD11c^+^ CD11b^−^), T cells (CD3^+^ CD56^−^), cytotoxic T cells (CD3^+^ CD8^+^), memory cytotoxic T cells (CD3^+^ CD8^+^ CD45RO^+^), naive cytotoxic T cells (CD3^+^ CD8^+^ CD45RA^+^), Th cells (CD3^+^ CD4^+^), memory Th cells (CD3^+^ CD4^+^ CD45RO^+^), naive Th cells (CD3^+^ CD4^+^ CD45RA^+^), B cells (CD19^+^ CD3^−^), memory B cells (CD19^+^ CD27^+^), regulatory B cells (CD19^+^ CD5^+^) and natural killer cells (CD56^+^ CD3^−^). T0 represents the baseline when the participants were sober. Our objective was to identify within-group differences following binge drinking. Statistical comparisons were done using repeated measures one-way ANOVA with Dunnett *post hoc* testing. One, two, three, or four asterisks indicate the significance levels *p* < 0.05, *p* < 0.01, *p* < 0.001, and *p* < 0.0001. (n = 19).

The immunophenotyping results revealed significant changes in relative cell counts (%) (Figure 2). T cells demonstrated an increase at T2 (*p* = 0.0141), while memory cytotoxic T cells showed a substantial rise both at T1 (*p* < 0.0001) and T2 (*p* = 0.0212). Contrastingly, naive cytotoxic T cells were seen to decrease at T1 (*p* < 0.001) and T2 (*p* = 0.0303). Memory T helper (Th) cells also exhibited a significant increase at T1 (*p* = 0.009) and T2 (*p* = 0.0368), whereas naive Th cells displayed a decrease at T1 (*p* = 0.0179). Additionally, a significant reduction in natural killer (NK) cells was observed at both T1 (*p* = 0.0129) and T2 (*p* = 0.0069). The relative cell counts of granulocytes did not show any significant changes. Nevertheless, the results indicate acute effects of alcohol intoxication on peripheral blood leukocyte counts as early as 1 h and 2 h after exposure.

### 3.3 *In vitro* studies—whole blood incubation with 26.04 and 86.8 mmol/l ethanol

**FIGURE 3:**
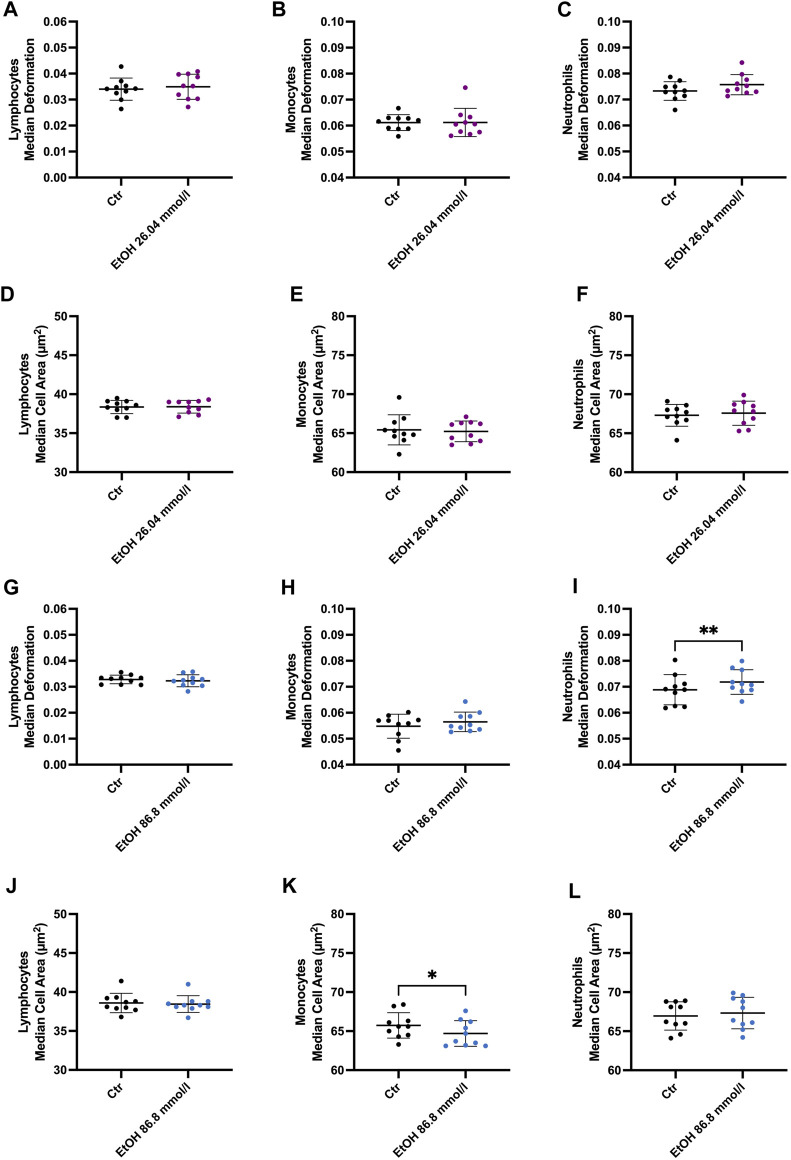
Whole blood incubation with 26.04 and 86.8 mmol/l ethanol. **(A–F)** Median deformation and median cell area of lymphocytes **(A)**, monocytes **(B)** and neutrophils **(C)** after incubation at 26.04 mmol/l. There were no significant changes after 2 h of incubation in ethanol. **(G–L)** Median deformation and median cell area of the 3 cell subsets incubated in 86.8 mmol/l ethanol for 2 h. Statistical comparisons were done using paired t-tests. One, two, three, or four asterisks indicate the significance levels *p* < 0.05, *p* < 0.01, *p* < 0.001, and *p* < 0.0001. (26.04 mmol/l, n = 10; 86.8 mmol/l n = 10).

Incubation of whole blood with 26.04 mmol/l ethanol did not result in any significant alterations in cell deformability, size, volume, or Young’s modulus in lymphocytes, monocytes, or neutrophils (Figure 3). Interestingly, the SD cell area of the lymphocytes was significantly higher compared to the control group (CTR) (3.966 ± 0.3456 µm^2^, CTR 3.754 ± 0.3454 µm^2^), indicating diverse responses of this heterogenous cell population to ethanol (Supplementary Figure S3). However, neutrophil deformability increased slightly at 26.04 mmol/l (Figure 3C) and was significantly elevated at 86.8 mmol/l (0.0718 ± 0.0047 compared to CTR 0.0686 ± 0.0058, *p* = 0.0097, Figure 3I). At 86.8 mmol/l the median Young’s modulus of neutrophils was significantly lower (0.992 ± 0.0189 kPa, compared to CTR 1.011 ± 0.0264 kPa, *p* = 0.0052), indicating a reduction of cell stiffness (Supplementary Figure S4I). Furthermore, the cell size (64.7 ± 1.64 µm^2^ compared to CTR 65.73 ± 1.62, *p* = 0.0247, Figure 3K), Young’s modulus (1.067 ± 0.03 kPa, compared to CTR1.118 ± 0.064 kPa, Supplementary Figure S4H) and volume (295.2 ± 10.44 µm^3^ compared to CTR 306.2 ± 12.25 µm^3^, *p* = 0.0111, Supplementary Figure S4N) of monocytes were significantly decreased at 86.8 mmol/l.

### 3.4 *In vitro* stimulation of whole blood with LPS and CytoStim™ in the presence of 26.04 or 86.8 mmol/l ethanol

**FIGURE 4:**
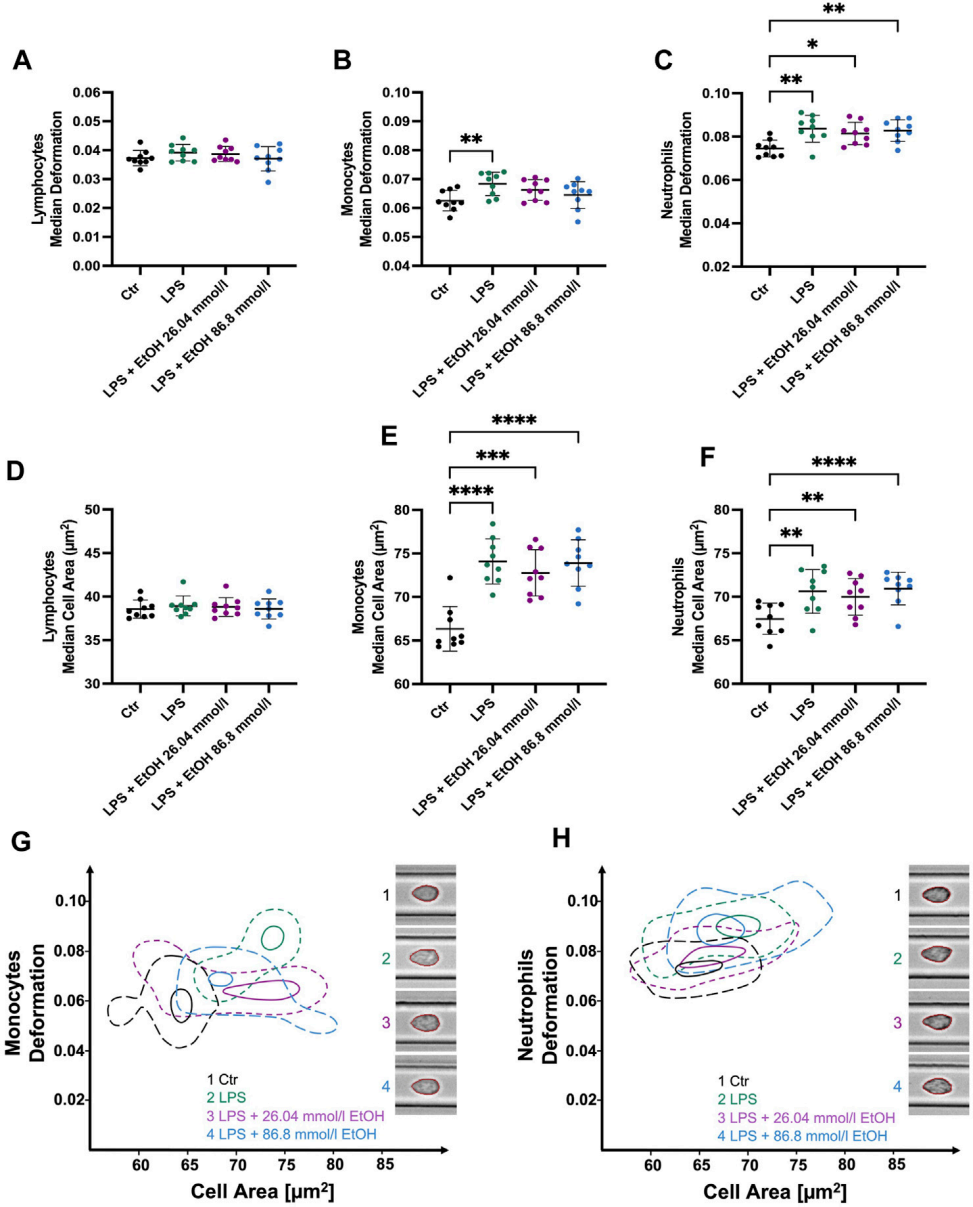
Whole blood stimulation with LPS in the presence of 26.04 and 86.8 mmol/l ethanol. **(A–C)** Median cell deformation. **(D–F)** Median cell area (µm^2^) of lymphocytes, monocytes, and neutrophils. No significant differences in lymphocyte deformability **(A)** and size **(D)** were observed after stimulation with LPS. LPS-stimulated monocytes **(B, E)** and neutrophils **(C, F)** exhibit significantly increased deformability and cell size compared to unstimulated cells. Monocyte stimulation in the presence of either 26.04 mmol/l or 86.8 mmol/l ethanol did not result in significant changes in deformability. Whereas stimulated neutrophils and those co-incubated with ethanol showed a significantly higher deformability and cell size. **(G–H)** Contour plots of monocytes **(G)** and neutrophils **(H)** illustrating the differences among the experimental conditions, ranging from untreated control cells, through LPS-stimulated cells, to cells co-incubated with either 26.04 mmol/l or 86.8 mmol/l ethanol, respectively. Statistical comparisons were done using repeated measures one-way ANOVA with Dunnett *post hoc* testing. One, two, three, or four asterisks indicate the significance levels *p* < 0.05, *p* < 0.01, *p* < 0.001, and *p* < 0.0001. (n = 9).

In parallel to our *ex vivo* approach, we aimed to determine the influence of ethanol on cell morphological and mechanical characteristics during cell activation *in vitro*. While LPS stimulation did not result in any significant changes to the biophysical characteristics of lymphocytes, as shown in Figure 4A; Supplementary Figure S5, a significant increase in the SD cell area was observed (4.37 ± 0.46 µm^2^ compared to CTR 4.14 ± 0.47 µm^2^, *p* = 0.0278). Furthermore, a significant increase in cell deformability in monocytes was observed following LPS stimulation (0.0684 ± 0.004 compared to CTR 0.0626 ± 0.004, *p* = 0.0089, Figure 4B), whereas no such effect was apparent when ethanol was added (0.0662 ± 0.004). LPS-stimulated monocytes and those co-incubated with ethanol had a significantly higher cell area and volume (Figures 4E, G; Supplementary Figure S5N). The Young’s modulus was found to be higher in stimulated and ethanol-exposed monocytes, reflecting an increase in cell stiffness. However, these changes were found to be significant only in cells co-incubated with the higher ethanol concentration (Young’s modulus 1.104 ± 0.021 kPa compared to CTR 1.042 ± 0.023 kPa, *p* = 0.0008, Supplementary Figure S5H). Similar results were obtained with respect to neutrophil size and volume, with both parameters being significantly increased during LPS stimulation as well as in co-incubation with ethanol (Figures 4F, H; Supplementary Figure S5O). Interestingly, unlike monocytes, neutrophils displayed a significant increase in deformability both in the presence and absence of ethanol (Figure 4C). In addition, a decrease in neutrophils Young’s modulus was observed which was significant in co-incubation with 26.04 mmol/l ethanol (0.953 ± 0.0188 kPa compared to CTR 0.974 ± 0.019, *p* = 0.04, Supplementary Figure S5I). Hence, neutrophils became softer during the LPS stimulation, while monocytes showed an increase in cell rigidity, especially in the presence of a higher ethanol concentration (Supplementary Figures S5H, I).

**FIGURE 5:**
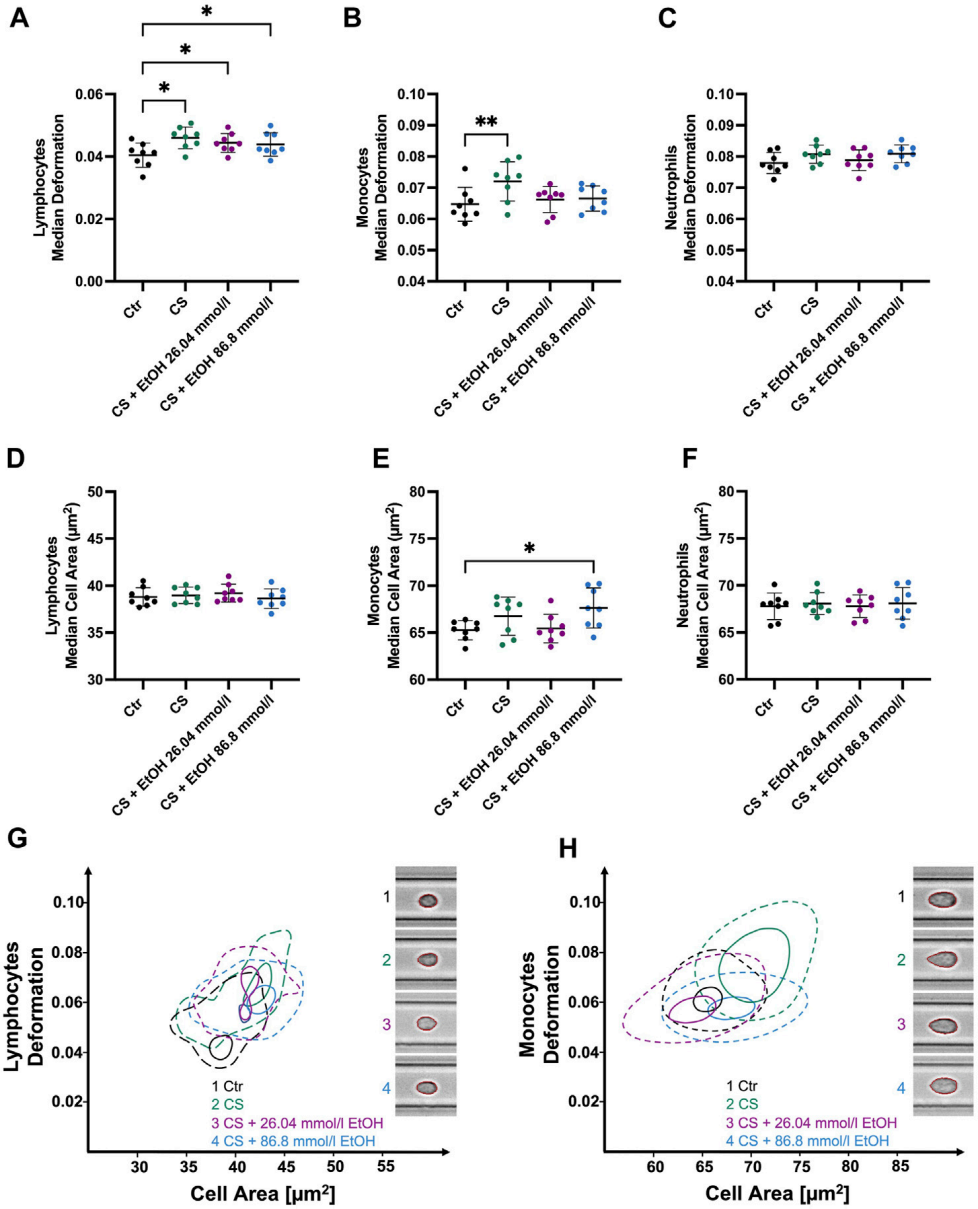
Whole blood stimulation with CytoStim™ (CS) in the presence of 26.04 and 86.8 mmol/l ethanol. **(A–C)** ∆ median cell deformation **(D–F)** median cell area (µm^2^) of lymphocytes, monocytes, and neutrophils. CS-stimulated lymphocytes and monocytes were significantly more deformable but showed no significant changes in cell size compared to non-stimulated cells. However, ethanol presence had no significant effect on the deformability of stimulated lymphocytes **(A)**. Whereas stimulated monocytes co-incubated with either 26.04 mmol/l or 86.8 mmol/l did not show a significant increase in deformability compared to the control group. CS had no significant effects on the size or deformability of neutrophils. **(G–H)** Contour plots of lymphocytes **(G)** and monocytes **(H)** highlight the differences between the experimental conditions. Statistical comparisons were done using repeated measures one-way ANOVA with Dunnett *post hoc* testing. One, two, three, or four asterisks indicate the significance levels *p* < 0.05, *p* < 0.01, *p* < 0.001, and *p* < 0.0001. (n = 8).

CytoStim™ stimulation led to significantly more deformable lymphocytes (0.046 ± 0.003 compared to CTR 0.04 ± 0.004, *p* = 0.0253, Figure 5A). Notably, lymphocytes co-incubated with 26.04 mmol/l (median 0.0443 ± 0.003, *p* = 0.0474) or 86.8 mmol/l (median 0.0439 ± 0.0037, *p* = 0.0253) ethanol during the stimulation process did also display a significant increase in deformability compared to the control group. In contrast, no significant changes in lymphocyte cell size were detected in response to CytoStim™ (Figure 5D). However, monocytes stimulated with CytoStim™ showed similar results compared to those stimulated with LPS. While stimulation in the absence of ethanol led to significantly elevated deformability (0.0721 ± 0.0063 compared to CTR 0.065 ± 0.005 *p* = 0.0052, Figure 5B), co-incubation with either 26.04 mmol/l or 86.8 mmol/l ethanol did not result in significant changes compared to the control group. It is worth noting that, unlike the LPS-induced increase in monocyte size, no such changes were detected with CytoStim™ as a stimulant.

Moreover, our analysis could not detect any significant changes in neutrophil deformability, size, Young’s modulus, or volume. However, stimulated neutrophils displayed significantly increased SD deformation (0.0148 ± 0.002 compared to CTR 0.012 ± 0.0008, *p* = 0.0061), SD cell area (5.33 ± 0.159 µm^2^ compared to CTR 5.11 ± 0.22 µm^2^, *p* = 0.0139) and SD Young’s modulus (0.154 ± 0.052 kPa compared to CTR 0.069 ± 0.007 kPa, *p* = 0.0078), again, indicating a heterogenous response within the neutrophil’s population (Supplementary Figure S6).

## 4 Discussion

Clinical and animal studies have revealed that alcohol consumption, particularly at acute or chronically high levels, not only increases the susceptibility to infections but also contributes to poorer outcomes and slower recovery in traumatic injuries such as burns or bone fractures (Schmidt and Lint, 1972; Saitz et al., 1997; Silver et al., 2008; Sears et al., 2011; Lauing et al., 2012; Shults et al., 2015; Shults et al., 2016). This is primarily attributed to alterations in critical immune cell functions in both innate and adaptive immunity.

Recent research has focused on the morphological and mechanical characteristics of cells as potential biomarkers for various diseases. It is hypothesized that specific disease-related alterations in cells’ biophysical properties could be detected using methods such as RT-DC (Guck and Chilvers, 2013; Otto et al., 2015). Up to now, a few approaches have been made by characterizing the blood cells in patients with viral respiratory tract and Epstein-Barr-virus (EBV) infections (Toepfner et al., 2018), COVID-19 patients (Kubánková et al., 2021) and recently, singularized suspended cells from the human colon for differentiating between healthy and tumorous tissues (Soteriou et al., 2023). Interestingly, the distinct leukocyte subsets showed different cell mechanical responses, depending on the viral pathogen involved (Kubánková et al., 2021). These alterations primarily involved changes in cell size and deformability. The successful identification and validation of specific cell mechanical profiles could pave the way for incorporating these parameters into diagnostic applications or as biomarkers.

Ethanol, a small amphiphilic molecule, interacts with biological membranes in various ways. It predominantly localizes within the headgroup region of the lipids, where it can form hydrogen bonds with the phosphate and carbonyl groups (Rottenberg, 1992; Feller et al., 2002; Stetter and Hugel, 2013). This interaction contributes to altered membrane structures, potentially leading to observed effects on membrane properties, such as an increase in permeability, fluidity, and elasticity (Chin and Goldstein, 1977; Komatsu and Okada, 1997; Ly and Longo, 2004). Furthermore, ethanol can interact with membrane-associated proteins such as ion channels, receptors, adhesion molecules and enzymes, causing structural changes, and influencing vital cell functions (Escriba et al., 2008; Tóth et al., 2014). Additionally, evidence suggests that the cytoskeleton—a complex network of microtubules, actin, and intermediate filaments—may also be vulnerable to ethanol toxicity (Yoon et al., 1998; Tomás et al., 2003; Tomás et al., 2005; Fletcher and Mullins, 2010; Tóth et al., 2014; Adhikari et al., 2023). However, most of these findings are derived from either animal or membrane model studies and their comparability to *in vivo* or *ex vivo* studies might be limited. Our findings demonstrate that a single episode of binge drinking does not impact the biophysical cell properties of *ex vivo* analyzed steady-state lymphocytes, monocytes, or neutrophils within the first 2 h post-binge drinking. It is important to note that this study, conducted using RT-DC, focused on deformability, Young’s modulus, cell size, and volume, while membrane fluidity and permeability were not investigated. Consequently, further research is needed to explore these biophysical parameters that have shown alterations in previous studies.

In line with prior studies, immunophenotyping results revealed significantly altered relative cell counts of leukocyte subtypes. Several studies demonstrated a disbalance between naive and memory CD4 and CD8 T cells, resulting in a shift toward a memory phenotype (Cook et al., 1994; Cook et al., 1995; Song et al., 2002). Higher numbers of memory T cells have been linked to chronic inflammatory disorders, autoimmunity and pathologies that primarily occur in older individuals (Hakim and Gress, 2007; Chou and Effros, 2013). Further, it is suggested that the observed shift in T cell phenotype in patients with alcohol use disorder (AUD) is analogous to that observed in immunosenescence (Zuluaga et al., 2020). Additionally, we observed a significant decrease in relative NK cell counts, which has been described for early patients with AUD (Perney et al., 2003). Conversely, patients with alcohol-related cirrhosis seem to have an increased NK cell percentage with elevated cytotoxicity, compared to healthy individuals (Sehgal et al., 2020). Such discrepancies might be a result of different stages of liver disease highlighting the time and dose dependency of alcohol-induced alterations in the immune system (Zuluaga et al., 2022). Consistent with prior research, we did not observe significant alterations in B cell subtypes, monocytes, or granulocytes within the first 2 h after binge drinking (Afshar et al., 2015; Haag et al., 2022).

Our *in vitro* studies yielded results similar to the *ex vivo* measurements with steady-state leukocytes. Apart from a change in the SD cell area of lymphocytes, no significant alterations in biophysical parameters were observed in leukocytes co-incubated with 26.04 mmol/l (1.2‰) ethanol. In our investigation, we noticed a significant increase in neutrophil cell deformability and the SD cell area, alongside a decrease in neutrophils and monocytes Young’s modulus, indicating an overall reduction in cell stiffness. Previous research, utilizing a membrane model, deduced that the localization of ethanol molecules in the headgroup area of membranes leads to an expansion of the headgroup area, causing an overall thinning of the membrane. This enlarged area diminishes the interactions between these headgroups, which subsequently leads to a reduced Young’s modulus (Stetter and Hugel, 2013). While these observations were conducted in considerably higher ethanol concentrations, ranging between 10% and 40%, our findings propose that such effects might be discernible even at lower concentrations.

In our study, we aimed to learn about ethanol’s influence on the biophysical cell properties of leukocytes during the stimulation process. LPS is detected by PRR and prompts an innate immune response via the toll-like receptor (TLR)-4 complex, especially in monocytes and neutrophils (Ciesielska et al., 2021). As anticipated, we observed no significant alterations in the biophysical properties of the lymphocyte population, attributable to their lack of responsiveness to short-time LPS exposure (Chaiwut and Kasinrerk, 2022). Interestingly, LPS stimulation led to a significant change in SD cell area of lymphocytes, implicating a heterogenous response to LPS in the lymphocyte population. Notably, NK cells, which are known to respond to LPS are also classified within the lymphocyte population in RT-DC measurements and may cause this observation (Kanevskiy et al., 2013; Otto et al., 2015). Consistent with prior research, monocytes exhibited significantly increased deformability upon LPS stimulation and decreased compressibility, as evidenced by an increase in Young’s modulus (Ravetto et al., 2014). The observed effects in monocytes after LPS exposure may indicate two potential processes: cytoskeletal reorganization for migration and differentiation into macrophages or dendritic cells (Vicente-Manzanares and Sánchez-Madrid, 2004; Jakubzick et al., 2017). Indeed, studies have noted alterations in elastic properties during cell differentiation, specifically an increase in cell stiffness (Darling et al., 2008; Yu et al., 2010). Remarkably, higher deformability was not evident when cells were co-incubated in ethanol. *In vitro* studies demonstrated that exposure of human monocytes to 25 mmol/l ethanol reduces the production of TLR 4-mediated proinflammatory cytokines such as tumor necrosis factor (TNF)-alpha in response to LPS, while it increases the anti-inflammatory cytokine IL-10 (Szabo et al., 1996). Additionally, it initiates a signal cascade, involving the heat shock transcription factor (HSF)-1, leading to the downregulation of TLR-4 (Mandrekar et al., 2008). It is well established that internalization and downregulation of TLR 4 effectively terminate the pro-inflammatory immune reaction (Ciesielska et al., 2021). We hypothesize that the previously described anti-inflammatory effects of ethanol, and in turn the absence of a pro-inflammatory response, impede the reorganization of the cytoskeleton. The underlying mechanisms might be pleiotropic, considering the variability in membrane constitution among different subpopulations and the reported effects of ethanol on the cell membrane proteins, the bilayer itself, transcription factors, and the cytoskeleton (Tóth et al., 2014; Poggi et al., 2015).

LPS stimulation also resulted in a significant increase in neutrophil deformability, cell area and volume. Unlike monocytes, these responses were not hampered by co-incubation with ethanol, underscoring the heterogenous response of immune cells to ethanol. Recent research observed enhanced priming and ROS production in isolated neutrophils following binge alcohol consumption in healthy volunteers, while others report the opposite effects (Stadlbauer et al., 2019; Haag et al., 2022). Notably, both studies found an increase in non-phagocytosing cells. In our study, the stimulated as well as co-incubated cells demonstrated similar significant changes compared to the control group. Hence, further research is required to investigate a potential correlation between the phagocytosis capacity and biophysical parameters and whether these can be determined via RT-DC.

In this study, we observed a significant increase in lymphocyte cell deformability and a decrease in cell stiffness during stimulation with CytoStim™. As APCs, such as monocytes, are critical for T cell stimulation with CytoStim™, we hypothesize that the observed increase in monocyte deformability indicates their involvement in this process. Moreover, there is evidence suggesting that IFN-gamma, produced by Th cells 1, NK cells, and CD8 T cells, can independently activate monocytes and polarize them toward a highly pro-inflammatory macrophage phenotype, possibly resulting in the observed altered biophysical properties (Luque-Martin et al., 2021). In contrast to the changes observed during lymphocyte activation, monocytes did not display increased deformability when co-incubated with ethanol. This observation gains importance considering that *in vitro* studies have reported a disruption in the antigen presentation abilities of monocytes when exposed to ethanol, resulting in inhibited superantigen-induced T cell proliferation (Szabo et al., 1993; Szabo et al., 1995). Interestingly, ethanol co-incubation did not hamper the increase in lymphocyte deformability, raising the question of whether impaired antigen presentation by monocytes and lymphocyte proliferation might be detectable via RT-DC at later time points in the stimulation process. In contrast to the stimulation with LPS, no similar increase in cell size was observed in monocytes when stimulated with CytoStim™. This intriguing discrepancy may be related to an apoptotic response triggered in monocytes by CytoStim™. Past research has demonstrated that the superantigen staphylococcal enterotoxin B (SEB) induces apoptosis in monocytes (Takahashi et al., 2001). Interestingly, this apoptotic response was inhibited when LPS was present (Takahashi et al., 2001). As CytoStim™ operates similarly to SEB, it might also induce apoptosis and thereby prevent the increase in cell size that we observed with LPS stimulation.

While our study provides meaningful insights into the impact of ethanol on the biophysical properties of immune cells, it is important to acknowledge several limitations associated with this being a pilot study. One significant limitation is the small sample size which reduces the statistical power and generalizability of our results. Another notable limitation is the lack of a follow-up phase; the potential effects of alcohol intoxication on cell mechanical properties might be detectable at later time points, beyond those measured in our study. A follow-up phase would have also allowed us to monitor the duration of the observed changes in immune cell counts and to determine when the phenotype reverts to its normal state. For instance, the effects were transient in certain studies and faded within hours (Stadlbauer et al., 2019; Haag et al., 2022). Our study design did not account for these time-dependent changes. This calls for future studies to incorporate time-course analysis with a higher sample size for a more comprehensive understanding of cellular kinetics post-ethanol exposure. Our examination primarily focused on cellular populations that are heterogeneous in nature, encompassing multiple cell types with contrasting functions within the immune system. This heterogeneity necessitates a more detailed characterization of each individual cell population. For instance, the significance of RT-DC measurements could be enhanced by applying the recently developed fluorescence module, allowing better discrimination between different cell types while characterizing the cell mechanical properties (Rosendahl et al., 2018). Furthermore, we did not perform immunophenotyping on the samples from our *in vitro* experiments. Future studies could contrast *ex vivo* and *in vitro* immunophenotyping results to investigate whether the observed effects are directly induced by ethanol or if there are additional *in vivo* factors at play. Given the scope of this pilot study, we did not examine the expression of activation markers, cytokines, or changes in actin polymerization, which limits our understanding of the functional implications of the observed biophysical changes.

In conclusion, our study suggests that a single episode of binge drinking does not significantly affect the biophysical properties of steady-state leukocytes in healthy individuals. However, it does lead to significant changes in the relative cell counts and a shift toward a memory T cell phenotype. On the other hand, when cells were stimulated, monocytes showed a susceptibility to ethanol, manifested as a hindered increase in cell deformability. This indicates that ethanol might inhibit the reorganization of the cytoskeleton during the stimulation process. Additional studies are necessary to confirm our observations, further assess the presence of ethanol-specific cell mechanical profiles in both acute and chronic exposure, and explore the potential of biophysical properties as biomarkers or prognostic indicators in alcohol-related health damage.

## Data Availability

The raw data supporting the conclusion of this article will be made available by the authors, without undue reservation.

## Abbreviations

AFM, atomic force microscopy; APC, antigen-presenting cell; AUD, alcohol use disorder; AUDIT, alcohol use disorder identification test; BAC, blood alcohol concentration; CD, cluster of differentiation; EBV, Epstein-Barr virus; EDTA, ethylene diamine tetra acetic acid; FACS, fluorescence activated cell sorting; HSF, heat shock transcription factor; IFN, interferon; LPS, lipopolysaccharide; MHC, major histocompatibility complex; NK, natural killer; PBS, phosphate-buffered saline; PRR, pathogen-recognition receptor; RBC, red blood cell; ROI, region of interest; ROS, reactive oxygen species; RT-DC, real-time deformability cytometry; SEB, *staphylococcus* enterotoxin B; SD, standard deviation; TBW, total body water; TCR, T cell receptor; Th, T helper; TLR, toll-like receptor; TNF, tumor necrosis factor.
