# Supplementary material for: Pilot study on the influence of acute alcohol exposure on biophysical parameters of leukocytes
Source: Front Mol Biosci. 2023 Aug 8;10:1243155. doi: 10.3389/fmolb.2023.1243155 (PMC10442941; doi:10.3389/fmolb.2023.1243155)
Supplement: Supplementary file 1 [file DataSheet1.pdf]

Supplementary Material

Pilot study on the influence of acute alcohol exposure on biophysical parameters of leukocytes

Puya Shalchi-Amirkhiz[1], Tristan Bensch[1], Undine Proschmann[1], Ann-Kathrin Stock[2,3], Tjalf Ziemssen[1,]† and Katja Akgün[1,] †

[1] Multiple Sclerosis Center, Center of Clinical Neuroscience, Department of Neurology, University Hospital Carl Gustav Carus, Dresden University of Technology, Dresden, Germany

[2] Cognitive Neurophysiology, Department of Child and Adolescent Psychiatry, Faculty of Medicine of the TU Dresden, Dresden, Germany

[3] Biopsychology, Department of Psychology, School of Science, TU Dresden, Dresden, Germany

† These authors contributed equally to this work.

*** Correspondence:**
PD Dr. Katja Akgün
Katja.Akguen@uniklinikum-dresden.de

1 Supplementary Figures

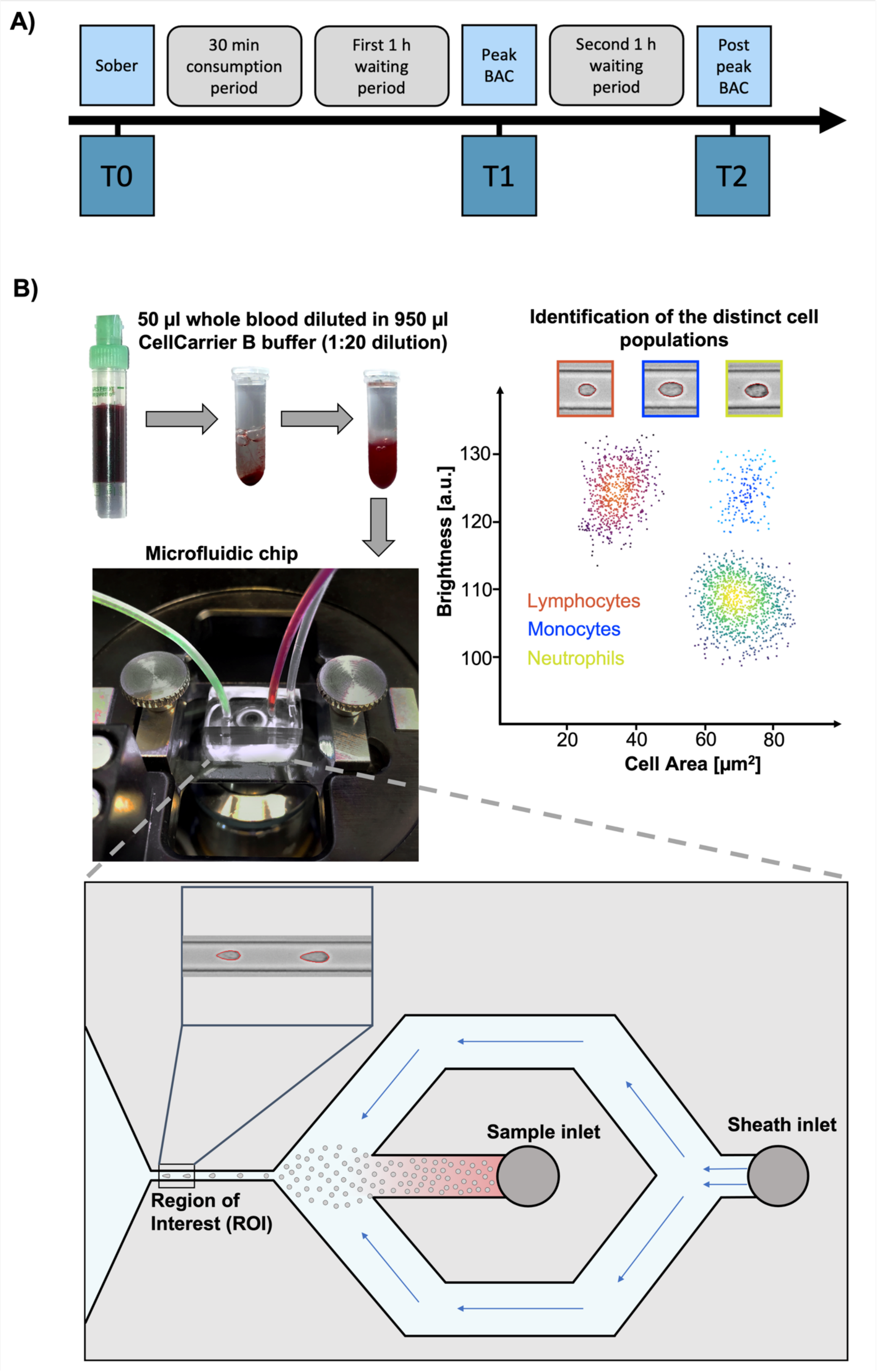
A)
Sober
30 min consumption period
First 1 h waiting period
Peak BAC
Second 1 h waiting period
Post peak BAC
T0
T1
T2
B)
50 µl whole blood diluted in 950 µl CellCarrier B buffer (1:20 dilution)
Identification of the distinct cell populations
Microfluidic chip
Brightness [a.u.]
130
120
110
100
Lymphocytes
Monocytes
Neutrophils
20
40
60
80
Cell Area [µm²]
Sample inlet
Sheath inlet
Region of Interest (ROI)

Supplementary Figure 1. A) Study scheme. Blood was collected and alcohol breath tests were performed at three different time points (T0, T1, T2). T0 represents the baseline when the participants were sober. Then they had 30 min to consume their alcoholic beverage. An hour later, they reached their peak BAC, which was marked as T1. After a second waiting period, the post-peak BAC was measured (T2). Our objective was to identify within-group differences following binge drinking. For data analysis, we employed a repeated measures one-way ANOVA, followed by Dunnett post hoc testing. **B) RT-DC measurement scheme.** Blood was collected into a sodium citrate S-monovette (Sarstedt). Subsequently, 50 µl of the blood sample was diluted with 950 µl CellCarrier B measurement buffer and mixed gently by rotating the sample tube. The sample was then aspirated into the PEEK tubes and connected to the microfluidic chip. During the measurement, the high-speed camera was focused on the region of interest (ROI). For analysis, the cells were gated in the brightness–area parameter plot.

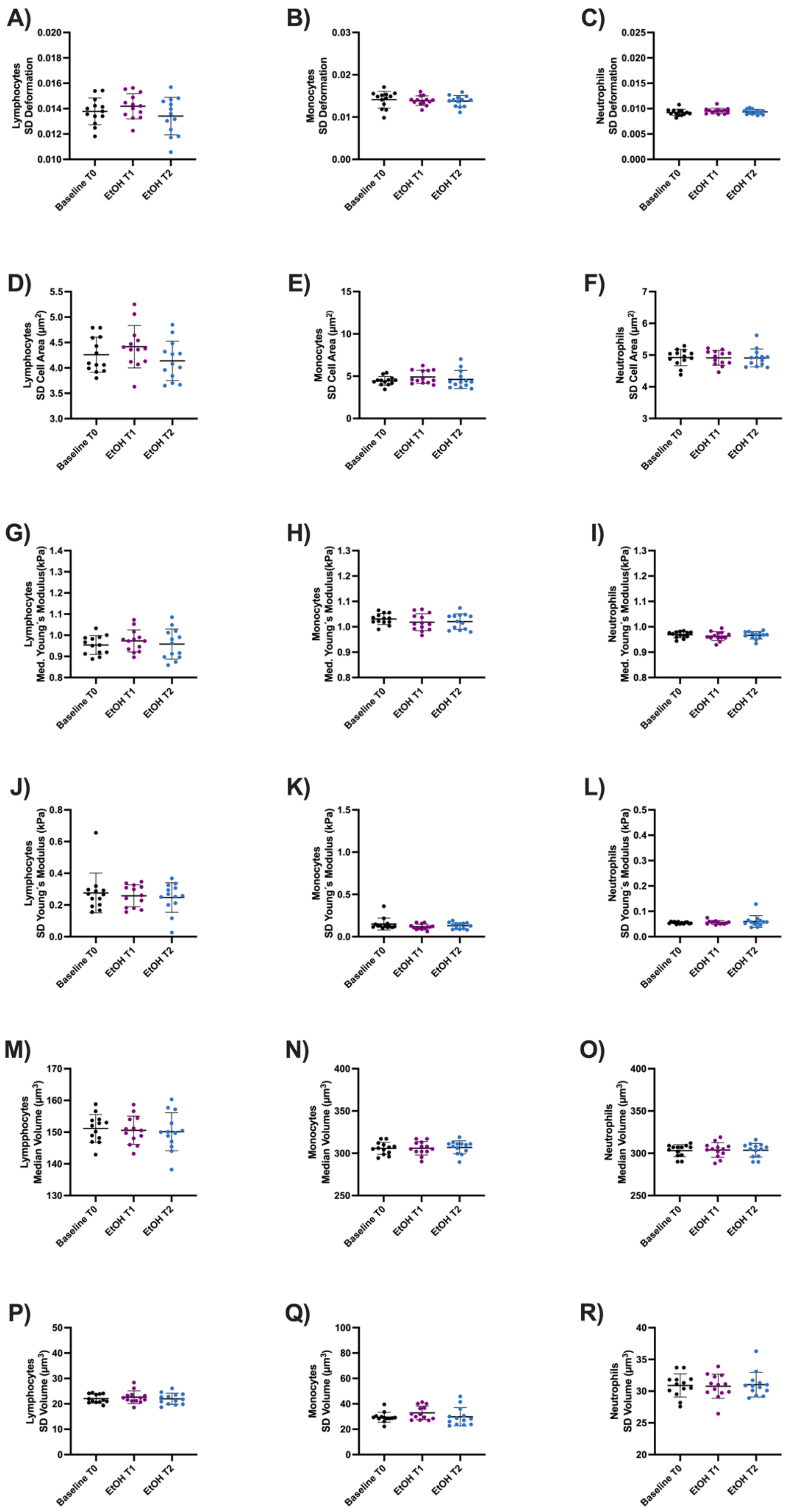
A)
Lymphocytes SD Deformation
B)
Monocytes SD Deformation
C)
Neutrophils SD Deformation
D)
Lymphocytes SD Cell Area (µm²)
E)
Monocytes SD Cell Area (µm²)
F)
Neutrophils SD Cell Area (µm²)
G)
Lymphocytes Med. Young´s Modulus(kPa)
H)
Monocytes Med. Young´s Modulus(kPa)
I)
Neutrophils Med. Young´s Modulus(kPa)
J)
Lymphocytes SD Young´s Modulus (kPa)
K)
Monocytes SD Young´s Modulus (kPa)
L)
Neutrophils SD Young´s Modulus (kPa)
M)
Lympphocytes Median Volume (µm³)
N)
Monocytes Median Volume (µm³)
O)
Neutrophils Median Volume (µm³)
P)
Lymphocytes SD Volume (µm³)
Q)
Monocytes SD Volume (µm³)
R)
Neutrophils SD Volume (µm³)
Baseline T0
EtOH T1
EtOH T2

Supplementary Figure 2. **Effect of binge drinking on cell mechanical properties. A) - C)** SD Deformation of lymphocytes (A), monocytes (B) and neutrophils (C) before (T0), 1 h (T1) and 2 h (T2) after intoxication with an alcoholic beverage. **D) – F)** SD cell area (μm^2) **G) – H)** Median Young´s modulus (kPa) **J) – L)** SD Young´s modulus (kPa) **M) – O)** Median Volume (μm^3) **P) – R)** SD Volume (μm^3). Statistical comparisons were done using repeated measures one-way ANOVA with Dunnett post hoc testing. One, two, three, or four asterisks indicate the significance levels $p < 0.05$, $p < 0.01$, $p < 0.001$, and $p < 0.0001$. ($n = 13$)

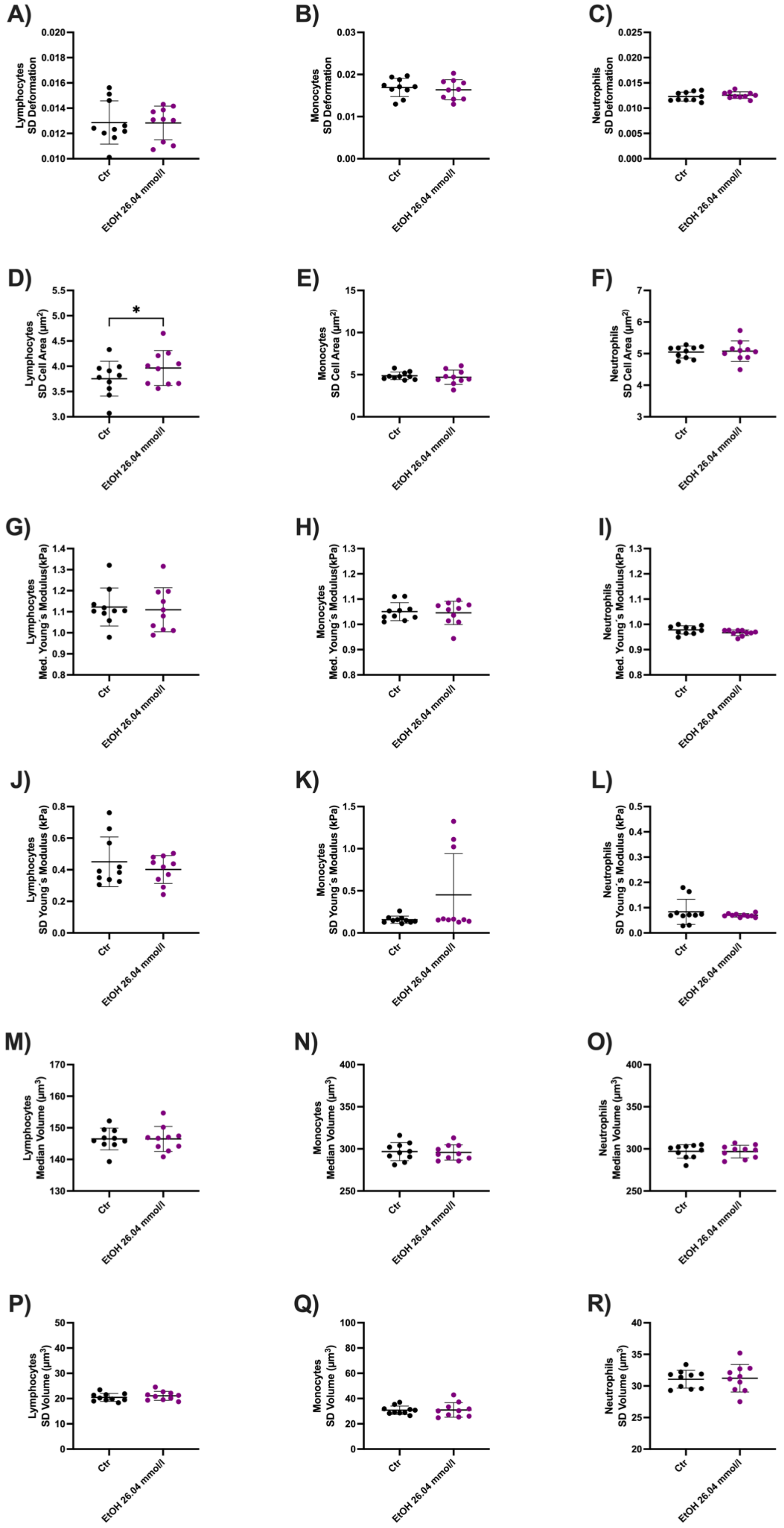
A)
Lymphocytes SD Deformation
0.020
0.018
0.016
0.014
0.012
0.010
Ctr
EtOH 26.04 mmol/l
B)
Monocytes SD Deformation
0.03
0.02
0.01
0.00
Ctr
EtOH 26.04 mmol/l
C)
Neutrophils SD Deformation
0.025
0.020
0.015
0.010
0.005
0.000
Ctr
EtOH 26.04 mmol/l
D)
Lymphocytes SD Cell Area (µm²)
*
5.5
5.0
4.5
4.0
3.5
3.0
Ctr
EtOH 26.04 mmol/l
E)
Monocytes SD Cell Area (µm²)
15
10
5
0
Ctr
EtOH 26.04 mmol/l
F)
Neutrophils SD Cell Area (µm²)
7
6
5
4
3
Ctr
EtOH 26.04 mmol/l
G)
Lymphocytes Med. Young's Modulus(kPa)
1.4
1.3
1.2
1.1
1.0
0.9
0.8
Ctr
EtOH 26.04 mmol/l
H)
Monocytes Med. Young's Modulus(kPa)
1.3
1.2
1.1
1.0
0.9
0.8
Ctr
EtOH 26.04 mmol/l
I)
Neutrophils Med. Young's Modulus(kPa)
1.3
1.2
1.1
1.0
0.9
0.8
Ctr
EtOH 26.04 mmol/l
J)
Lymphocytes SD Young's Modulus (kPa)
0.8
0.6
0.4
0.2
0.0
Ctr
EtOH 26.04 mmol/l
K)
Monocytes SD Young's Modulus (kPa)
1.5
1.0
0.5
0.0
Ctr
EtOH 26.04 mmol/l
L)
Neutrophils SD Young's Modulus (kPa)
0.5
0.4
0.3
0.2
0.1
0.0
Ctr
EtOH 26.04 mmol/l
M)
Lymphocytes Median Volume (µm³)
170
160
150
140
130
Ctr
EtOH 26.04 mmol/l
N)
Monocytes Median Volume (µm³)
400
350
300
250
Ctr
EtOH 26.04 mmol/l
O)
Neutrophils Median Volume (µm³)
400
350
300
250
Ctr
EtOH 26.04 mmol/l
P)
Lymphocytes SD Volume (µm³)
50
40
30
20
10
0
Ctr
EtOH 26.04 mmol/l
Q)
Monocytes SD Volume (µm³)
100
80
60
40
20
0
Ctr
EtOH 26.04 mmol/l
R)
Neutrophils SD Volume (µm³)
40
35
30
25
20
Ctr
EtOH 26.04 mmol/l

Supplementary Figure 3. **Whole blood incubation with 26.04 mmol/l ethanol. A) - C)** SD Deformation of lymphocytes (A), monocytes (B) and neutrophils (C) after incubation with 26.04 mmol/l ethanol. **D) – F)** SD cell area (µm^2) **G) – H)** Median Young´s modulus (kPa) **J) – L)** SD Young´s modulus (kPa) **M) – O)** Median Volume (µm^3) **P) – R)** SD Volume (µm^3). Statistical comparisons were done using paired t-tests. One, two, three, or four asterisks indicate the significance levels $p < 0.05$, $p < 0.01$, $p < 0.001$, and $p < 0.0001$. (26.04 mmol/l, n = 10)

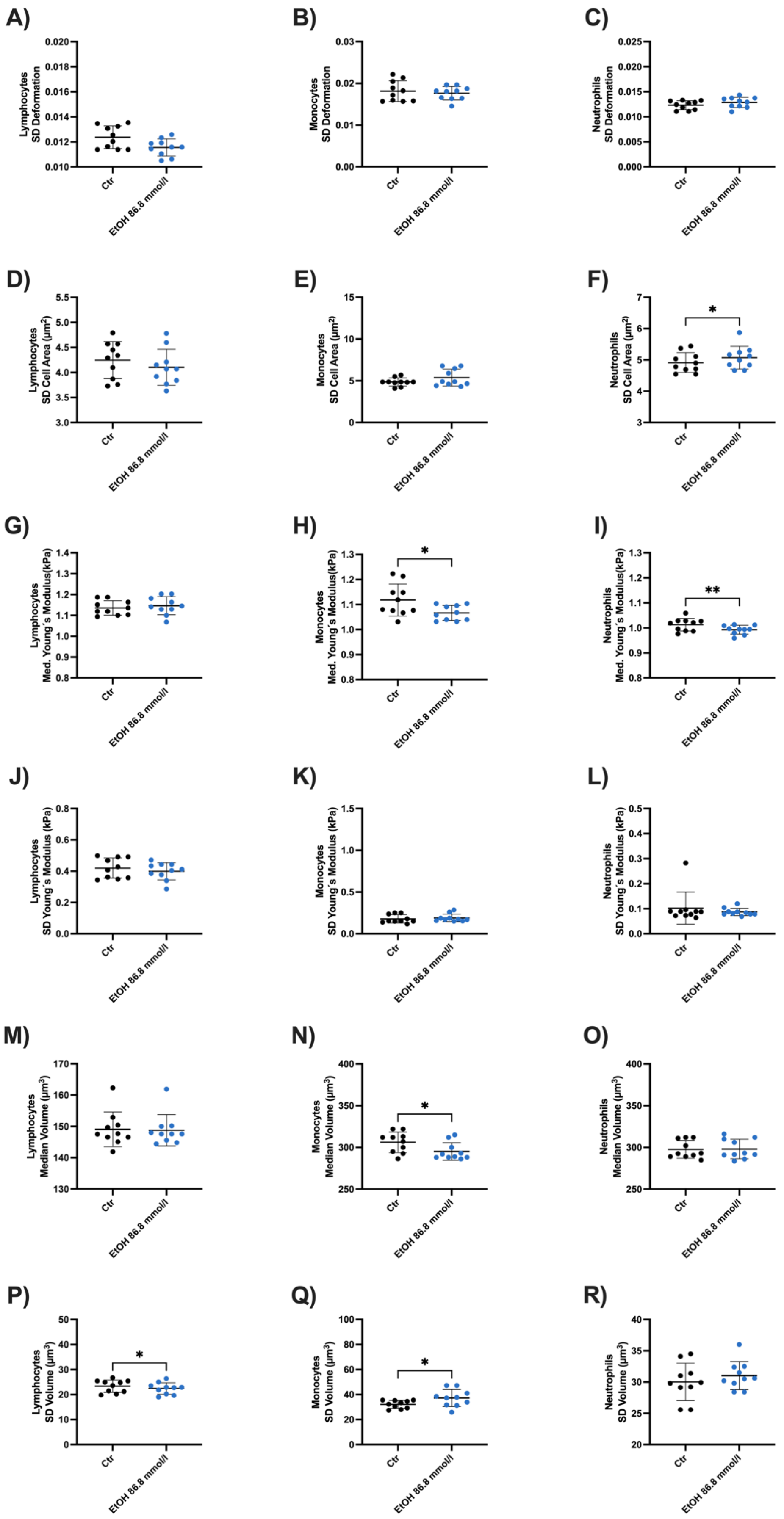
A)
Lymphocytes SD Deformation
B)
Monocytes SD Deformation
C)
Neutrophils SD Deformation
D)
Lymphocytes SD Cell Area (µm²)
E)
Monocytes SD Cell Area (µm²)
F)
Neutrophils SD Cell Area (µm²)
G)
Lymphocytes Med. Young´s Modulus(kPa)
H)
Monocytes Med. Young´s Modulus(kPa)
I)
Neutrophils Med. Young´s Modulus(kPa)
J)
Lymphocytes SD Young´s Modulus (kPa)
K)
Monocytes SD Young´s Modulus (kPa)
L)
Neutrophils SD Young´s Modulus (kPa)
M)
Lymphocytes Median Volume (µm³)
N)
Monocytes Median Volume (µm³)
O)
Neutrophils Median Volume (µm³)
P)
Lymphocytes SD Volume (µm³)
Q)
Monocytes SD Volume (µm³)
R)
Neutrophils SD Volume (µm³)
Ctr
EtOH 86.8 mmol/l

Supplementary Figure 4. **Whole blood incubation with 86.8 mmol/l ethanol. A) - C)** SD Deformation of lymphocytes (A), monocytes (B) and neutrophils (C) after incubation with 86.8 mmol/l ethanol. **D) – F)** SD cell area (μm^2) **G) – H)** Median Young´s modulus (kPa) **J) – L)** SD Young´s modulus (kPa) **M) – O)** Median Volume (μm^3) **P) – R)** SD Volume (μm^3). Statistical comparisons were done using paired t-tests. One, two, three, or four asterisks indicate the significance levels $p < 0.05$, $p < 0.01$, $p < 0.001$, and $p < 0.0001$. (86.8 mmol/l n = 10)

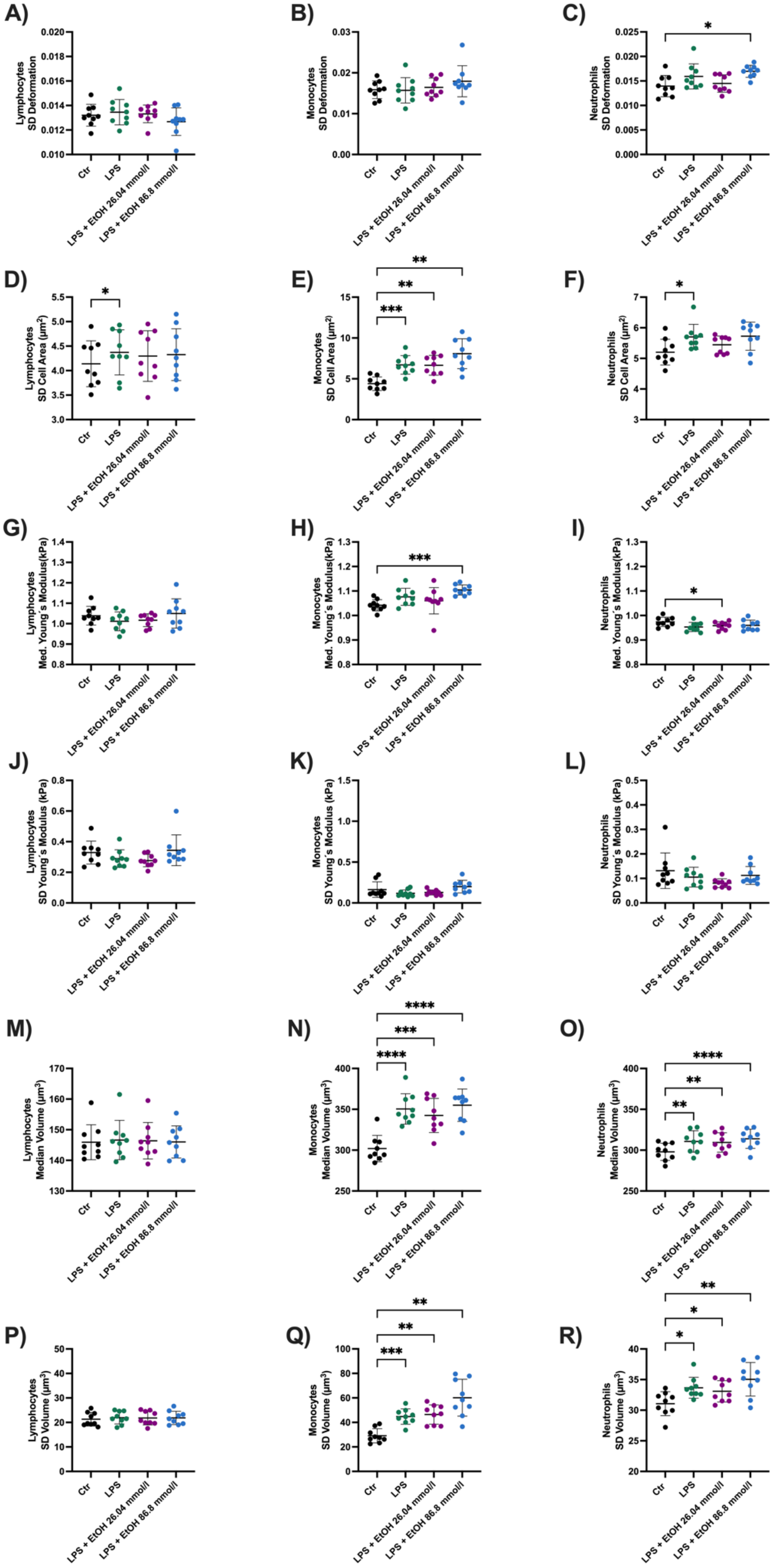
A)
Lymphocytes SD Deformation
B)
Monocytes SD Deformation
C)
Neutrophils SD Deformation
D)
Lymphocytes SD Cell Area (µm²)
E)
Monocytes SD Cell Area (µm²)
F)
Neutrophils SD Cell Area (µm²)
G)
Lymphocytes Med. Young's Modulus(kPa)
H)
Monocytes Med. Young's Modulus(kPa)
I)
Neutrophils Med. Young's Modulus(kPa)
J)
Lymphocytes SD Young's Modulus (kPa)
K)
Monocytes SD Young's Modulus (kPa)
L)
Neutrophils SD Young's Modulus (kPa)
M)
Lymphocytes Median Volume (µm³)
N)
Monocytes Median Volume (µm³)
O)
Neutrophils Median Volume (µm³)
P)
Lymphocytes SD Volume (µm³)
Q)
Monocytes SD Volume (µm³)
R)
Neutrophils SD Volume (µm³)
Ctr
LPS
LPS + EtOH 26.04 mmol/l
LPS + EtOH 86.8 mmol/l

Supplementary Figure 5. **Whole blood stimulation with LPS in the presence of 26.04 and 86.8 mmol/l ethanol. A) - C)** SD Deformation of lymphocytes (A), monocytes (B) and neutrophils (C). **D) – F)** SD cell area (μm^2) **G) – H)** Median Young´s modulus (kPa) **J) – L)** SD Young´s modulus (kPa) **M) – O)** Median Volume (μm^3) **P) – R)** SD Volume (μm^3). Statistical comparisons were done using repeated measures one-way ANOVA with Dunnett post hoc testing. One, two, three, or four asterisks indicate the significance levels $p < 0.05$, $p < 0.01$, $p < 0.001$, and $p < 0.0001$. ($n = 9$)

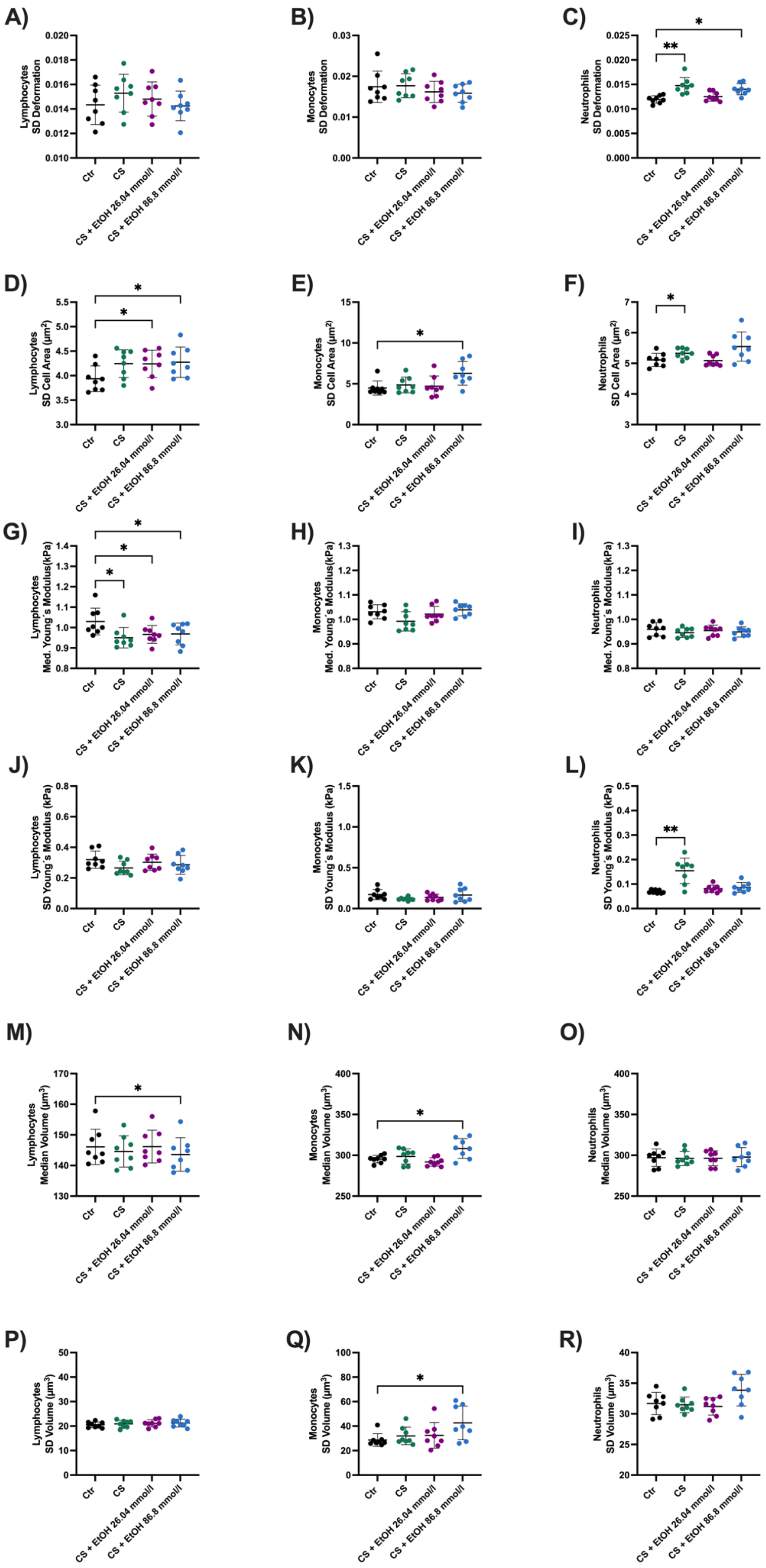
A)
Lymphocytes SD Deformation
B)
Monocytes SD Deformation
C)
Neutrophils SD Deformation
D)
Lymphocytes SD Cell Area (µm²)
E)
Monocytes SD Cell Area (µm²)
F)
Neutrophils SD Cell Area (µm²)
G)
Lymphocytes Med. Young's Modulus(kPa)
H)
Monocytes Med. Young's Modulus(kPa)
I)
Neutrophils Med. Young's Modulus(kPa)
J)
Lymphocytes SD Young's Modulus (kPa)
K)
Monocytes SD Young's Modulus (kPa)
L)
Neutrophils SD Young's Modulus (kPa)
M)
Lymphocytes Median Volume (µm³)
N)
Monocytes Median Volume (µm³)
O)
Neutrophils Median Volume (µm³)
P)
Lymphocytes SD Volume (µm³)
Q)
Monocytes SD Volume (µm³)
R)
Neutrophils SD Volume (µm³)
Ctr
CS
CS + EtOH 26.04 mmol/l
CS + EtOH 86.8 mmol/l

Supplementary Figure 6. **Whole blood stimulation with Cytostim® in the presence of 26.04 and 86.8 mmol/l ethanol. A) - C)** SD Deformation of lymphocytes (A), monocytes (B) and neutrophils (C). **D) – F)** SD cell area (μm^2) **G) – H)** Median Young´s modulus (kPa) **J) – L)** SD Young´s modulus (kPa) **M) – O)** Median Volume (μm^3) **P) – R)** SD Volume (μm^3). Statistical comparisons were done using repeated measures one-way ANOVA with Dunnett post hoc testing. One, two, three, or four asterisks indicate the significance levels $p < 0.05$, $p < 0.01$, $p < 0.001$, and $p < 0.0001$. (n = 8)

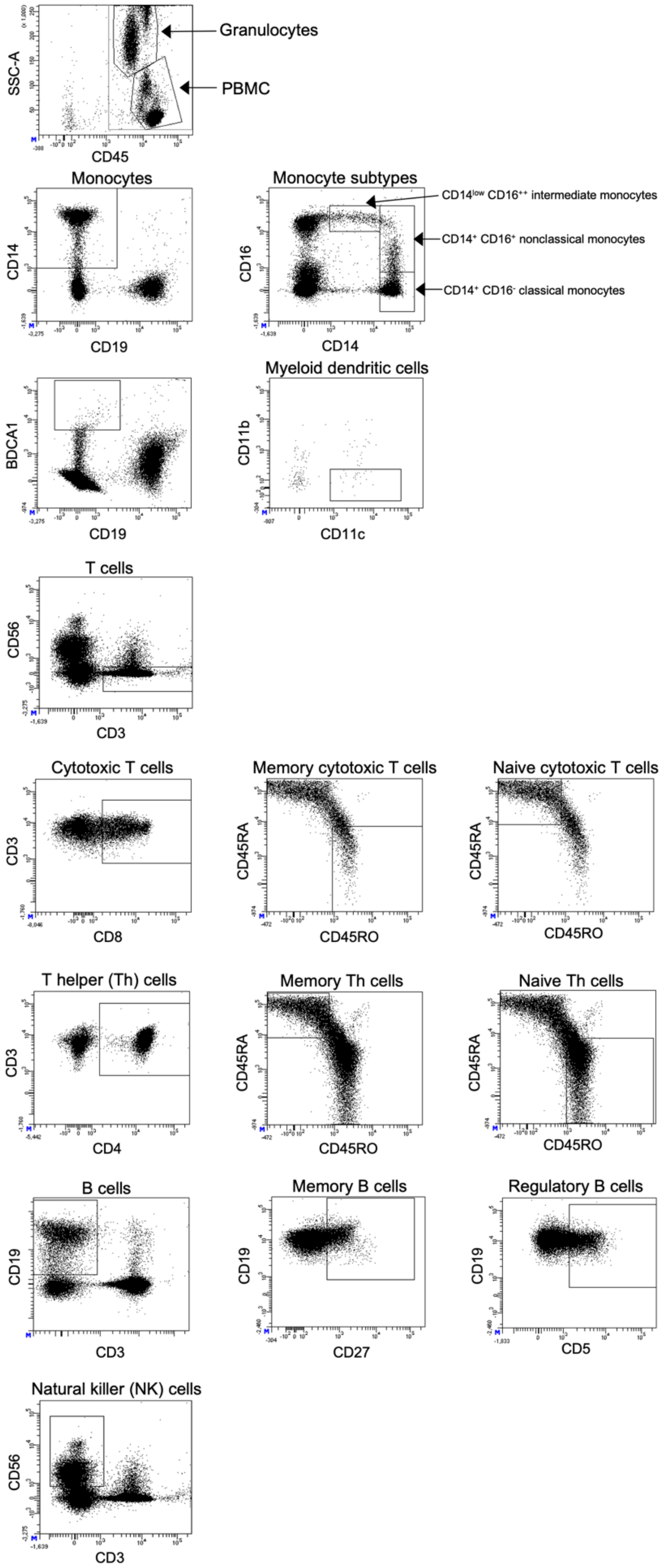
Granulocytes
PBMC
SSC-A
CD45
Monocytes
CD14
CD19
Monocyte subtypes
CD14low CD16++ intermediate monocytes
CD14+ CD16+ nonclassical monocytes
CD14+ CD16- classical monocytes
CD16
CD14
BDCA1
CD19
Myeloid dendritic cells
CD11b
CD11c
T cells
CD56
CD3
Cytotoxic T cells
CD3
CD8
Memory cytotoxic T cells
CD45RA
CD45RO
Naive cytotoxic T cells
CD45RA
CD45RO
T helper (Th) cells
CD3
CD4
Memory Th cells
CD45RA
CD45RO
Naive Th cells
CD45RA
CD45RO
B cells
CD19
CD3
Memory B cells
CD19
CD27
Regulatory B cells
CD19
CD5
Natural killer (NK) cells
CD56
CD3

Supplementary Figure 7. Gating strategy of analyzed peripheral immune cell subsets evaluated by FACS analysis. The following cell subsets were included: PBMCs, granulocytes, monocytes ($CD14^{+}$), classical monocytes ($CD14^{+}$ $CD16^{-}$), nonclassical monocytes ($CD14^{+}$ $CD16^{+}$), intermediate monocytes ($CD14^{low}$ $CD16^{+}$), myeloid dendritic cells ($BDCA1^{+}$ $CD11c^{+}$ $CD11b^{-}$), T cells ($CD3^{+}$ $CD56^{-}$), cytotoxic T cells ($CD3^{+}$ $CD8^{+}$), memory cytotoxic T cells ($CD3^{+}$ $CD8^{+}$ $CD45RO^{+}$), naive cytotoxic T cells ($CD3^{+}$ $CD8^{+}$ $CD45RA^{+}$), Th cells ($CD3^{+}$ $CD4^{+}$), memory Th cells ($CD3^{+}$ $CD4^{+}$ $CD45RO^{+}$), naive Th cells ($CD3^{+}$ $CD4^{+}$ $CD45RA^{+}$), B cells ($CD19^{+}$ $CD3^{-}$), memory B cells ($CD19^{+}$ $CD27^{+}$), regulatory B cells ($CD19^{+}$ $CD5^{+}$) and natural killer cells ($CD56^{+}$ $CD3^{-}$).
